# Robot-assisted laparoscopic repair of perineal hernia after abdominoperineal resection: A case report and review of the literature

**DOI:** 10.1016/j.ijscr.2018.12.009

**Published:** 2019-01-17

**Authors:** Pooya Rajabaleyan, Allan Dorfelt, Peiman Poornoroozy, Per Vadgaard Andersen

**Affiliations:** Department of Surgery A, Odense University Hospital, Sdr. Boulevard 29, Entrance 18, Penthouse Floor 2, 5000, Odense C, Denmark

**Keywords:** Perineal hernia, Robotic-assisted laparoscopic repair, Synthetic mesh, Abdominoperineal resection, Extralevator abdominoperineal excision, Case report

## Abstract

**Introduction:**

Perineal hernia is a protrusion of the pelvic floor containing intra-abdominal viscera. The occurrence of postoperative perineal hernia after abdominoperineal resection (APR) is rare, but reports have indicated a recent increase in occurrence following surgical treatment for rectal cancer. This has been attributed to a shift towards extralevator abdominoperineal resection, together with more frequent and long-term use of neoadjuvant therapy.

**Presentation of case:**

Here, we report the case of a patient who underwent APR for cancer. Twenty months postoperative, a perineal hernia was detected. The patient was electively scheduled for surgery. Robot-assisted laparoscopy was performed using the da Vinci Surgical System. The perineal hernia was repaired by primary closure with the placement of Symbotex Composite mesh as reinforcement for the pelvic floor. The surgery was performed without any adverse events, and the patient was discharged the day after surgery. Clinical follow-up proceeded at the designated time intervals without difficulties.

**Discussion:**

Recurrence rates of perineal hernia remain high, and surgeons face numerous challenges related to poor view, suturing and mesh placement in the deep pelvis. Numerous approaches have been described, but there is still no consensus as to the optimal repair technique for perineal hernia.

**Conclusion:**

Symptomatic perineal hernias can feasibly be repaired with robot-assisted laparoscopy. Furthermore, suturing and mesh placement require less effort with the robot approach when compared to the open and laparoscopic approaches. These promising findings are demonstrated in the included video.

## Highlights

• Incidence of perineal hernia might be on the rise due to modification of operative technique and probably increased usage of neoadjuvant therapy.
• Numerous approaches have been described for the repair of the defect, but none have yet been accepted as “Gold standard”.
• To our knowledge this is the first ever documented robot-assisted repair of a perineal hernia.
• In-depth description of operative method.
• Video demonstrating the ease of mesh placement and suturing in the deep pelvis.

## 1 Introduction

Perineal hernia is defined as a defect in the pelvic floor resulting in a bulging of intra-abdominal content through the perineum [1]. Secondary perineal hernias may occur as a rare complication after abdominoperineal resection (APR) in patients with rectal cancers [2,3]. Postoperative perineal hernia was first described by Yeoman in 1939 [4]. The incidence of perineal hernias after conventional APR is reported to be <1% [[5], [6], [7]]; however, two larger studies by West et al. [8] and Sayers et al. [9] reviewed the frequency of perineal hernias after extralevator abdominoperineal excision (ELAPE) and reported frequencies of 2.8% and 26%, respectively. The first was a heterogeneous multi-centre study consisting of 176 patients, with variable use of neoadjuvant radiochemotherapy and synchronous perineal defect repair, all of which could influence the risk of postoperative perineal hernia occurrence [8]. The second is the largest, consecutive, single-centre case series to date, which consisted of 54 patients [9]. At present, there is insufficient data to represent the true incidence of perineal hernia post-ELAPE. Although ELAPE has been shown to improve the postoperative oncological outcome, it leaves a significantly greater perineal wound when compared to APR due to the wider circumferential resection margin of the rectum [2,8]. This increases the rate of wound complications, and potentially contributes to perineal hernia formation [9,10]. In addition, neoadjuvant therapy is becoming more common, which may prevent the wound from healing sufficiently [[11], [12], [13]].

In general, perineal hernias proceed asymptomatically, which unfortunately results in a large number of unreported cases [6,10]. Perineal hernias without symptoms are usually treated conservatively. However, they can cause symptoms such as bulging with discomfort, which, when complicated by urinary dysfunction, intestinal obstruction or skin erosion, may represent an indication for surgical treatment [2,11].

Numerous approaches for surgical repair of the defect have been described in the literature, including open, laparoscopic or combined methods [2]. These are coupled with various techniques for repairing the perineal defect [2,11].

To our knowledge, our case report is the first to present the use of a robot-assisted approach to repair a secondary perineal hernia.

This work has been reported in line with the SCARE criteria [14].

## 2 Presentation of case

A 70-year old male with a history of ulcerative colitis was referred with a tumour in the ascending colon. He underwent laparoscopic intersphincteric proctocolectomy with a permanent ileostomy. The tumour was postoperatively staged as T4N1M0. The patient underwent adjuvant chemotherapy with 5-fluorouracil leucovorin and oxaliplatin (FOLFOX).

After 20 months, the patient returned with complaints of discomfort due to a bulge in the perineal region. During clinical examination in an upright position, a herniation was observed to be bulging out on the right side of the cicatrise in close proximity to the crenia ani, with no associated pain or compromised bowel function. The small intestine was visible through the skin. A computed tomography scan confirmed the presence of a perineal hernia containing the small intestine, and further excluded any recurrence of malignancy.

The robotic surgical da Vinci Si System was used. The abdomen was entered using an open technique, and a 12-mm balloon trocar for the robotic camera was placed above and to the right of the umbilicus. An additional three robotic ports were placed in the right lower quadrant, left lower quadrant and left upper quadrant. One 12-mm assisting port was placed above and between the two right robotic ports. The patient was placed in Trendelenburg’s position, tilted to the right, and the robot was docked from the patient’s left side over the left hip.

**Fig. 1:**
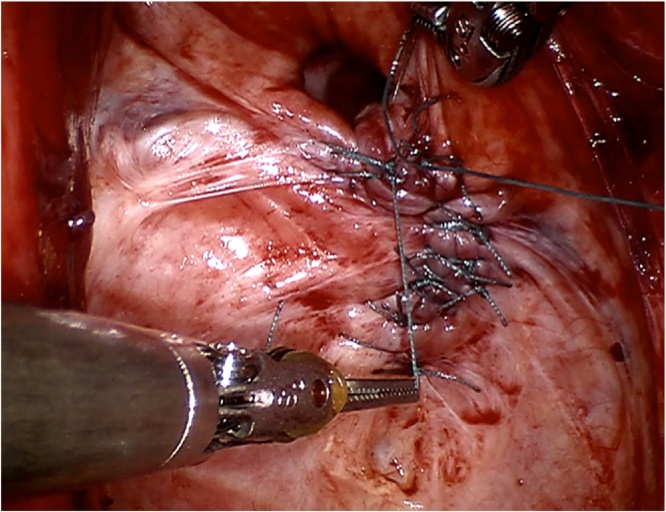
Most of the pelvic floor was closed with single knots, a small defect was left anterior to the urethra.

**Fig. 2:**
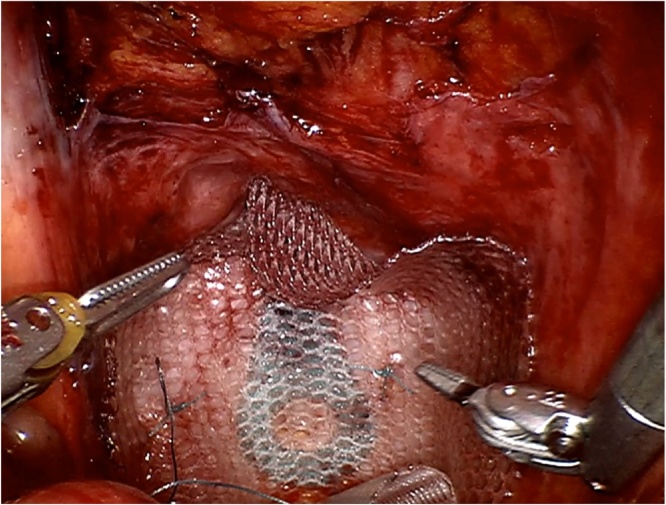
Mesh placement and subsequent fixation to the pelvic floor with single knots at 0, 90, 180 and 270°.

**Fig. 3:**
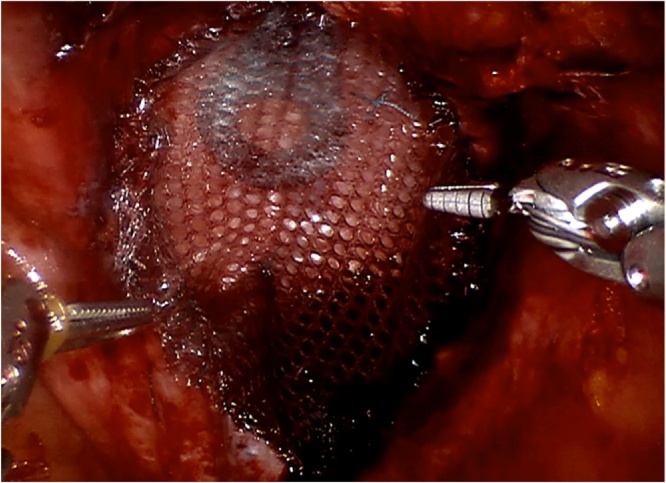
The edge of the mesh was approximated to the peritoneum with a running suture.

**Fig. 4:**
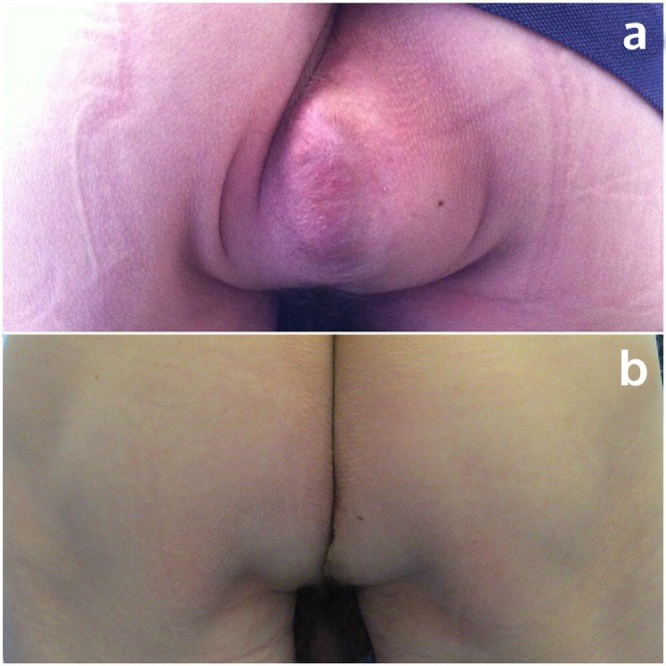
Image of the hernia (a) preoperatively and (b) postoperatively at 3 months follow-up.

Surgical exploration revealed an obvious defect in the pelvic floor approximately 6 cm in diameter, without any intra-abdominal adhesions. A significant part of the musculature of the pelvic floor was intact, and was closed with single knot Ethibond 2-0 sutures in a posterior to anterior direction, leaving a small defect anterior to the urethra (Fig. 1). The almost sealed defect was covered with a Symbotex Composite mesh (Covidien, Mansfield, MA, USA). The mesh was circular with a 12-cm diameter, and was sutured to the muscles of the pelvic floor with four Ethibond single knots at 0, 90, 180 and 270° (Fig. 2). Afterwards, four folds were cut in the mesh. The edge of the mesh was approximated to the peritoneum with running V-loc 3-0 sutures (Fig. 3). The procedure is illustrated in the included video. The patient was discharged the day after surgery without any *peri*- or postoperative complications. The first clinical follow-up examination occurred 3 weeks after the operation. Examination showed a clean wound with optimal healing. The Valsalva manoeuvre was performed while palpating the defect, with no palpable or observable bulging. At the next follow-up visit at 3 months postoperative, the patient reported no bulging and experienced no symptoms (Fig. 4). Upon examination, the wound was fully healed and there was no hernia recurrence.

## 3 Discussion

Postoperative perineal hernias continue to pose a challenge for surgeons. Recently, there have been significant developments in the surgical techniques used for rectal cancers, and surgeons are progressively leaning towards the radical ELAPE approach rather than conventional APR [8,9]. Moreover, neoadjuvant and adjuvant radio(chemo)therapy are becoming more common [11,12]. These modifications to treatments are collectively providing an improved oncological outcome; however, the incidence of perineal hernias might be increased as a consequence [8,11].

At present, the obstacles surgeons face when attempting to repair a perineal hernia include the successful closure of the defect while overcoming the anatomical complexity and strained overview of the pelvic floor. Suturing and mesh placement continue to be arduous tasks. Numerous approaches have been described for repair of the defect, but none have been accepted as the “Gold Standard”. Furthermore, there is a high recurrence rate. Mjoli et al. [10] reported a recurrence rate of 30% in their pooled analysis, which included 43 patients treated between 1944 and 2011. Balla et al. [12] reported a recurrence rate of 24% in their systematic review including 108 patients treated between 2012 and 2016. Exact data is not available due to the small numbers of patients reported in the literature.

Currently utilised approaches for perineal hernia closure include open surgery with perineal, abdominal or combined techniques and laparoscopic surgery with an abdominal approach [2,10]. The methods of repair vary, consisting of primary sutures, mesh placement, a combination of these methods or the use of a muscle flap [2,8]. Open perineal and abdominal approaches are more invasive, and consequently increase the risk for wounds and other complications; however, they allow satisfactory access for mesh placement and suturing. The perineal approach is preferred over open abdominal repair in uncomplicated perineal hernias as it provides adequate exposure and allows the resection of excess skin in case of significant bulging [10]. The open abdominal approach is favoured over the perineal approach for cases where the bowel needs to be dissected out, and it also provides an opportunity to detect and manage possible recurrences [10]. On the other hand, laparoscopic surgery is less invasive, provides improved visualisation and shortens the postoperative hospital stay [2,[15], [16], [17], [18]]. However, suturing can be demanding, as well as positioning of the mesh, which is crucial for a successful outcome.

Robot-assisted laparoscopic surgery combines the discussed advantages, including the ease of suturing, mesh positioning and access to hard-to-reach areas. Furthermore, it is minimally invasive and results in a shorter hospital stay. The da Vinci Surgical System combines all positive aspects of both the laparoscopic and open approaches, without exposing the patient to any of the discussed disadvantages.

The chosen method of repair is crucial to avoid relapse of the hernia. Primary closure has been reported to have a recurrence rate of 50%, which is reduced to 20% with placement of a mesh [10]. We decided to use the coated monofilament polyester Symbotex Composite mesh (Covidien, Mansfield, MA, USA), which was secured with Ethibond non-absorbable sutures. The mesh has a bio-absorbable layer to reduce the risk of fistulation and adherence to intra-abdominal structures.

To our knowledge, this is the first report of its kind to describe the use of robot-assisted laparoscopy to repair a perineal hernia. Relevant data regarding this subject could not be found in a literature search of the PubMed, Cochrane and Embase databases, hence making it difficult to compare experiences and findings.

## 4 Conclusion

The surgery was performed without any adverse events. The patient was discharged the day after surgery with no signs of complications. Clinical follow-up performed at 3 weeks and 3 months postoperative showed a satisfactory result. This case report is limited to only one patient. As a result of the muscle-sparing intersphincteric amputation performed during the primary operation, the executed procedure was manageable as the majority of the pelvic floor was still intact. The video demonstrates the feasibility of the robotic technique, emphasising the ease of mesh placement and suturing in the deep pelvis. The robotic system could potentially be applicable for more complex cases, which may arise in the future.

## Conflicts of interest

The authors declare they have no conflicts of interest.

## Sources of funding

The authors had no sources of funding for their research.

## Ethical approval

Our institution exempts ethical approval for case reports.

## Consent

Written informed consent was obtained from the patient for publication of this case report, accompanying images and video.

## Author contribution

Study conception and design: Pooya Rajabaleyan, Per Vadgaard Andersen, Peiman Poornoroozy.

Data acquisition: Pooya Rajabaleyan, Per Vadgaard Andersen, Allan Dorfelt.

Literature review: Pooya Rajabaleyan, Per Vadgaard Andersen.

Drafting of the manuscript: Pooya Rajabaleyan, Per Vadgaard Andersen.

Critical revision: Per Vadgaard Andersen, Alan Dorfelt, Peiman Poornoroozy.

## Registration of research studies

The study was registered at http://www.researchregistry.com. With ID Number: researchregistry4371.

## Guarantor

Pooya Rajabaleyan.

## Provenance and peer review

Not commissioned externally peer reviewed.

## References

[1] Moshcowitz A.V. (1916). Perineal hernia. Surg. Gynecol. Obstet..

[2] Goedhart-de Haan A.M.S., Langenhoff B.S., Petersen D., Verheijen P.M. (2016). Laparoscopic repair of perineal hernia after abdominoperineal excision. Hernia.

[3] Allen S.K., Schwab K., Day A., Singh-Ranger D., Rockall T.A. (2015). Laparoscopic repair of postoperative perineal hernia using a two-mesh technique. Colorectal Dis..

[4] Yeomans F.C. (1939). Levator hernia, perineal and pudendal. Am. J. Surg..

[5] So J.B., Palmer M.T., Shellito P.C. (1997). Postoperative perineal hernia. Dis. Colon Rectum.

[6] Aboian E., Winter D.C., Metcalf D.R., Wolff B.G. (2006). Perineal hernia after proctectomy: prevalence, risks, and management. Dis. Colon Rectum.

[7] Levic K., Von Rosen K., Bulut O., Bisgaard T. (2017). Low incidence of perineal hernia repair after abdominoperineal resection for rectal cancer. Dan. Med. J..

[8] West N.P., Anderin C., Smith K.J.E., Holm T., Quirke P. (2010). Multicentre experience with extralevator abdominoperineal excision for low rectal cancer. Br. J. Surg..

[9] Sayers A.E., Patel R.K., Hunter I.A. (2015). Perineal hernia formation following extralevator abdominoperineal excision. Colorectal Dis..

[10] Mjoli M., Sloothaak D.A.M., Buskens C.J., Bemelman W.A., Tanis P.J. (2012). Perineal hernia repair after abdominoperineal resection: a pooled analysis. Colorectal Dis..

[11] Martijnse I.S., Holman F., Nieuwenhuijzen G.A.P., Rutten H.J.T., Nienhuijs S.W. (2012). Perineal hernia repair after abdominoperineal rectal excision. Dis. Colon Rectum.

[12] Balla A., Batista Rodríguez G., Buonomo N., Martinez C., Hernández P., Bollo J. (2017). Perineal hernia repair after abdominoperineal excision or extralevator abdominoperineal excision: a systematic review of the literature. Tech. Coloproctol..

[13] Artioukh D.Y., Smith R.A., Gokul K. (2007). Risk factors for impaired healing of the perineal wound after abdominoperineal resection of rectum for carcinoma. Colorectal Dis..

[14] Agha R.A., Fowler A.J., Saeta A., Barai I., Rajmohan S., Orgill D.P. (2016). The SCARE statement: consensus-based surgical case report guidelines. Int. J. Surg..

[15] Abbas Y., Garner J. (2014). Laparoscopic and perineal approaches to perineal hernia repair. Tech. Coloproctol..

[16] Ghellai A.M., Islam S., Stoker M.E. (2002). Laparoscopic repair of postoperative perineal hernia. Surg. Laparosc. Endosc. Percutan. Tech..

[17] Ryan S., Kavanagh D.O., Neary P.C. (2010). Laparoscopic repair of postoperative perineal hernia. Case Rep. Med..

[18] Dulucq J.L., Wintringer P., Mahajna A. (2006). Laparoscopic repair of postoperative perineal hernia. Surg. Endosc..

